# Regulation of VEGFR Signalling in Lymphatic Vascular Development and Disease: An Update

**DOI:** 10.3390/ijms22147760

**Published:** 2021-07-20

**Authors:** Genevieve A. Secker, Natasha L. Harvey

**Affiliations:** Centre for Cancer Biology, University of South Australia and SA Pathology, Adelaide 5000, Australia; genevieve.secker@unisa.edu.au

**Keywords:** lymphangiogenesis, vascular endothelial growth factor (VEGF), vascular endothelial growth factor receptor (VEGFR), signal transduction, vascular development

## Abstract

The importance of lymphatic vessels in a myriad of human diseases is rapidly gaining recognition; lymphatic vessel dysfunction is a feature of disorders including congenital lymphatic anomalies, primary lymphoedema and obesity, while improved lymphatic vessel function increases the efficacy of immunotherapy for cancer and neurological disease and promotes cardiac repair following myocardial infarction. Understanding how the growth and function of lymphatic vessels is precisely regulated therefore stands to inform the development of novel therapeutics applicable to a wide range of human diseases. Lymphatic vascular development is initiated during embryogenesis following establishment of the major blood vessels and the onset of blood flow. Lymphatic endothelial progenitor cells arise from a combination of venous and non-venous sources to generate the initial lymphatic vascular structures in the vertebrate embryo, which are then further ramified and remodelled to elaborate an extensive lymphatic vascular network. Signalling mediated via vascular endothelial growth factor (VEGF) family members and vascular endothelial growth factor receptor (VEGFR) tyrosine kinases is crucial for development of both the blood and lymphatic vascular networks, though distinct components are utilised to different degrees in each vascular compartment. Although much is known about the regulation of VEGFA/VEGFR2 signalling in the blood vasculature, less is understood regarding the mechanisms by which VEGFC/VEGFD/VEGFR3 signalling is regulated during lymphatic vascular development. This review will focus on recent advances in our understanding of the cellular and molecular mechanisms regulating VEGFA-, VEGFC- and VEGFD-mediated signalling via VEGFRs which are important for driving the construction of lymphatic vessels during development and disease.

## 1. Introduction

**Figure 1:**
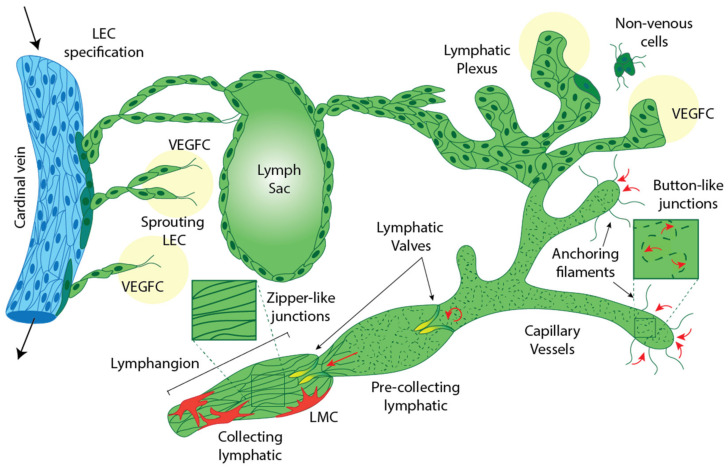
Lymphatic vessel development and structure. Lymphatic endothelial progenitor cells arise from venous and non-venous sources. VEGFC/VEGFR3 signalling promotes the sprouting and migration of PROX1-positive lymphatic endothelial cells (LECs) away from the veins to form an initial lymphatic plexus and lymph sacs. Continued sprouting and migration of LECs from initial lymphatic structures, as well as from non-venous sources, further elaborates the lymphatic vessel network. The mature lymphatic network comprises blind-ended initial vessels which display loose ‘button-like’ intercellular junctions and anchoring filaments which facilitate interstitial fluid uptake. Collecting lymphatic vessels display tight ‘zipper-like’ intercellular junctions and increased levels of surrounding extracellular matrix. Collecting lymphatic vessels are sub-divided into segments called lymphangions, punctuated by semi-lunar valves important for unidirectional lymph flow, and are invested with lymphatic muscle cells. Arrows indicate direction of flow.

The lymphatic vasculature comprises a hierarchical network of specialised vessels that work cooperatively to return interstitial fluid to the blood circulation and thereby maintain tissue fluid homeostasis. Lymphatic vessels are also crucial for regulating immunity and for the absorption of lipids and lipophilic molecules from the digestive tract [1,2,3]. In addition to these established roles of the lymphatic vasculature, exciting new physiological and pathological roles for lymphatic vessels are emerging [4,5], and the lymphatic vasculature is considered a promising route for more effective vaccine, immunotherapy and drug delivery [6,7]. While lymphatic vessels share some morphological similarities with blood vessels, their structure is exquisitely adapted to fulfil the unique functions of this vascular network. Blind-ended, initial lymphatic vessels are specialised to mediate fluid absorption. The endothelial cells that comprise initial lymphatics have a characteristic “oak leaf” shape, discontinuous, “button-like” intercellular junctions, low levels of extracellular matrix (ECM) at their basal aspect and a lack of mural cell association, all of which facilitate the influx of fluid, protein and cellular traffic (lymph) [8]. Lymph is transported from initial lymphatics via pre-collector vessels to larger collecting lymphatic vessels and, during this passage, is filtered through lymph nodes where it is presented to the immune system for surveillance. Lymph eventually returns to the blood circulation via connections between the right and thoracic lymphatic ducts with large veins in the jugular region [9]. Collecting lymphatic vessels are comprised of elongated endothelial cells with continuous “zipper-like” intercellular junctions, increased levels of ECM deposition and considerable lymphatic muscle cell investment, all of which promote lymph transport and prevent lymph leakage [8]. Specialised semi-lunar valves located in collecting lymphatic vessels facilitate unidirectional lymph flow and segment vessels into structural regions called lymphangions [10]. The recruitment of contractile lymphatic muscle cells to collecting vessels enables lymphangions to sequentially contract and relax in conjunction with valve opening and closing [11], a process that is regulated by both the autonomic nervous system [12,13] and the movement of surrounding muscles and tissues [4]. Together, these features of collecting lymphatic vessels propel lymph away from the periphery and return it to the bloodstream (Figure 1). Defects in any aspect of lymphatic vascular development can have major consequences on lymphatic function, resulting in disorders including vascular malformations, complex lymphatic vascular anomalies and primary lymphoedema, which can manifest either in utero, or later in life [4,5,14].

## 2. Roles of VEGFC, VEGFD and VEGFR3 Signalling during Lymphatic Vascular Development

In the mouse embryo, the first definitive sign that lymphatic vascular development has commenced is the onset of *Prox1* expression in a polarised population of cells within the cardinal and intersomitic veins at embryonic day (E) 9.5 [15]. PROX1-positive lymphatic endothelial progenitor cells bud and migrate from the veins in streams to form lymph sacs (also termed the ventral primordial thoracic duct) and a primary lymphatic plexus [15,16,17,18]. PROX1-positive lymphatic progenitor cells are also specified in the superficial venous plexus and primitive dermal capillary bed of the mouse embryo and contribute to generation of the superficial lymphatic plexus in the skin [18,19]. The exit of lymphatic endothelial progenitor cells from the veins is dependent on VEGFC [20], the VEGF family member most pivotal for lymphatic vascular development, together with the matrix protein CCBE1 [18,21,22] and protease ADAMTS3 [23,24], which together promote proteolytic cleavage of VEGFC to its maximally active form [25,26]. Mice deficient in *Vegfc* [20], *Ccbe1* [18,22] or *Adamts3* [23,24] exhibit profound oedema as a result of the arrest in lymphatic development, and die mid-gestation. While PROX1-positive cells are observed within the veins of these mutant mice, their exit is prohibited, illustrating that lymphatic endothelial progenitor cell specification is not dependent on VEGFC [18,19,20,22]. Recent work in both zebrafish and mice has shown that the number of progenitor cells specified in the veins is, however, amplified by VEGFC [19,27].

In contrast to the strict requirement for VEGFC during developmental lymphangiogenesis, VEGFD appears to be largely dispensable [28]. Specific roles for VEGFD have been identified during development of the initial lymphatic vessels in the skin of mice [29] and the facial lymphatics in zebrafish [30]. Intriguingly, the requirement of VEGFD for facial lymphangiogenesis in zebrafish appears to be dependent on signalling via VEGFR2 rather than VEGFR3 [31]. Recent work has provided deeper insight into the lymphatic vessel-specific roles of VEGFR3 during developmental lymphangiogenesis. While a crucial requirement for VEGFR3 during early cardiovascular development initially precluded assessment of the role of VEGFR3 in the lymphatic vasculature [32], characterisation of mice carrying mutations in the tyrosine kinase (Chy mice) [33], or ligand binding domains of VEGFR3 [34], revealed striking defects in embryonic lymphatic vascular development, demonstrating that VEGFR3 activity is required during developmental lymphangiogenesis. Recent work in which *Flt4* was selectively deleted in the lymphatic vasculature at the onset of lymphatic vascular development in the mouse embryo confirmed the crucial role of VEGFR3 in lymphatic endothelial cell sprouting and migration, demonstrating that VEGFR3 is the major VEGFC receptor driving developmental lymphangiogenesis [35]. Both VEGFC and VEGFD potently promote developmental and postnatal lymphangiogenesis; ectopic expression of either growth factor results in lymphatic vessel hyperplasia [36,37], and inhibition of VEGFC and VEGFD activity mediated via soluble VEGFR3 expression results in lymphatic vessel regression [38]. Intriguingly, while early studies suggested that, once established, the lymphatic vessels of most tissues in postnatal and adult mice are refractory to VEGFC and VEGFD blockade [39], recent work employing conditional deletion of *Vegfc*, and inducible expression of soluble VEGFR3, revealed that selected lymphatic beds including the intestinal lacteals and meningeal lymphatic vessels remain dependent on, or sensitive to, VEGFC/VEGFR3 signalling during adulthood [40,41].

## 3. The Vascular Endothelial Growth Factor Family

VEGF family members are essential to drive development of the blood and lymphatic vascular networks. There are five VEGF family members, VEGFA, VEGFB, VEGFC, VEGFD and placental growth factor (PIGF), which bind to their cognate receptors VEGFR1/VEGFR2/VEGFR3 as homodimers [37,42,43]. While VEGFA binds with high affinity to VEGFR1, it signals primarily via VEGFR2 to drive development of the blood vasculature [43]. In contrast, VEGFC and VEGFD are the primary growth factors regulating development of the lymphatic vasculature and predominantly signal via VEGFR3 [43]. There exists some crosstalk between these ligands and receptors; ectopic expression of VEGFA has been demonstrated to have pro-lymphangiogenic activity [44,45], while ectopic expression of VEGFD also promotes angiogenesis [46], and VEGFR3 plays important roles during embryonic blood vascular development and postnatal angiogenesis, despite being predominantly expressed in the lymphatic vasculature [47]. VEGFR2 and VEGFR3 also heterodimerise and respond to signalling induced by VEGFA and VEGFC [48,49], though the degree to which this interaction regulates distinct signalling outcomes in blood vessels and lymphatic vessels remains to be determined. Alternative splicing liberates a variety of VEGFA species which differ in their capacity to bind VEGFRs, co-receptors and ECM components [37,43]. In contrast, VEGFC and VEGFD comprise a central VEGF homology domain (VHD), flanked by N- and C-terminal pro-peptides that are proteolytically cleaved to generate mature growth factors with maximal affinity for binding to and activating VEGFR3 [50]. Proteases established to mediate VEGFC cleavage include ADAMTS3 [25], Adamts14 [51] (in zebrafish), plasmin [52], thrombin [53], kallikrein-related peptidase 3 (KLK3) [54] and cathepsin D [54]. While ADAMTS3, acting together with the matrix protein CCBE1, is crucial for physiological VEGFC processing and signalling during development, the requirement for plasmin, thrombin, KLK3 and cathepsin D for VEGFC activation in vivo remains to be determined. At least in the case of plasmin and thrombin, which are themselves activated in the setting of inflammation, these proteases are most likely to be important in settings of injury. VEGFD proteolysis is mediated by plasmin [52], thrombin [53], furin and proprotein convertases PC5 and PC7 [52,55]. While ADAMTS3 cleaves the N-terminal pro-peptide to activate VEGFC, it does not cleave VEGFD [23]. This distinction may underpin the crucial role of VEGFC, compared to VEGFD, during developmental lymphangiogenesis. Given that the activity of VEGFC and VEGFD is exquisitely regulated by factors including proteolytic activation and binding to ECM components, it follows that the precise spatial and temporal regulation of these cues during development is a determining factor in patterning the lymphatic vasculature. Two recent studies in zebrafish have demonstrated that this is the case; the first established that the secretion of type II collagen by notochord sheath cells is important for determining the route taken by lymphatic endothelial cells upon departure from the posterior cardinal vein (PCV) [56], and the second revealed that neurons and fibroblasts in the vicinity of the posterior cardinal vein provide localised sources of Adamts3, Adamts14, Ccbe1 and Vegfc that cumulatively activate Vegfc to establish the migration path taken by sprouting lymphatic endothelial cells [51]. Whether the effects of cleavage by different proteases mediate subtle differences in the magnitude of VEGFC and VEGFD initiated VEGFR3 signalling in vivo in mice or zebrafish is an interesting prospect and remains to be investigated.

## 4. Vascular Endothelial Growth Factor Receptors and Co-Receptors

VEGF family members predominantly bind to and activate the receptor tyrosine kinases, VEGFR1-3 [37,42,43]. These receptors share a similar overall structure, comprising an extracellular ligand-binding domain with seven immunoglobulin (Ig) homology domain repeats, a transmembrane domain and split tyrosine kinase domain. The first three Ig-like domains bind VEGF ligands, while domains 4–7 proximal to the membrane mediate receptor homodimerization (as well as VEGFR2/VEGFR3 heterodimerization) and activation [57]. While similar in structure, each of these receptors exhibit distinctions in their mechanisms of activation, signal transduction pathways and resultant biological effects [43]. In addition to binding VEGFRs, VEGF family members bind the co-receptors neuropilin 1 (NRP1), neuropilin 2 (NRP2) and heparan sulfate proteoglycans (HSPGs). In the vasculature, NRP1 is primarily restricted to arterial endothelium [58] and lymphatic vessel valves [59,60], where it acts as a semaphorin receptor to regulate valve development, whereas NRP2 is predominantly expressed in venous [58] and lymphatic endothelial cells [61]. NRP2 binds to and regulates VEGFC initiated signalling in the lymphatic vasculature [61]; *Nrp2^−/−^* mice exhibit pronounced defects in development of the superficial lymphatic vessels. CD146, also known as MCAM (melanoma cell adhesion molecule), was recently suggested to play a distinct role in transducing VEGFC-initiated sprouting signals in lymphatic endothelial cells. CD146 was documented to co-immunoprecipitate with VEGFC and VEGFR3 and, moreover, was demonstrated to directly bind VEGFC and transduce VEGFC-initiated signals via p38 kinase and ERK [62]. Knockdown of CD146 in zebrafish resulted in reduced parachordal lymphangioblast sprouting and interrupted formation of the thoracic duct [62]. The heparan sulfate proteoglycan syndecan-4 was recently shown to be the predominant HSPG in lymphatic endothelial cells and to interact with VEGFR3 following VEGFC treatment [63]. VEGFC-initiated VEGFR3 signalling was reduced in lymphatic endothelial cells depleted of syndecan-4, and *Sdc4^−/−^* mice were shown to exhibit defects in developmental and pathological lymphangiogenesis [63,64].

## 5. VEGFR Signal Transduction in Lymphatic Endothelial Cells

**Figure 2:**
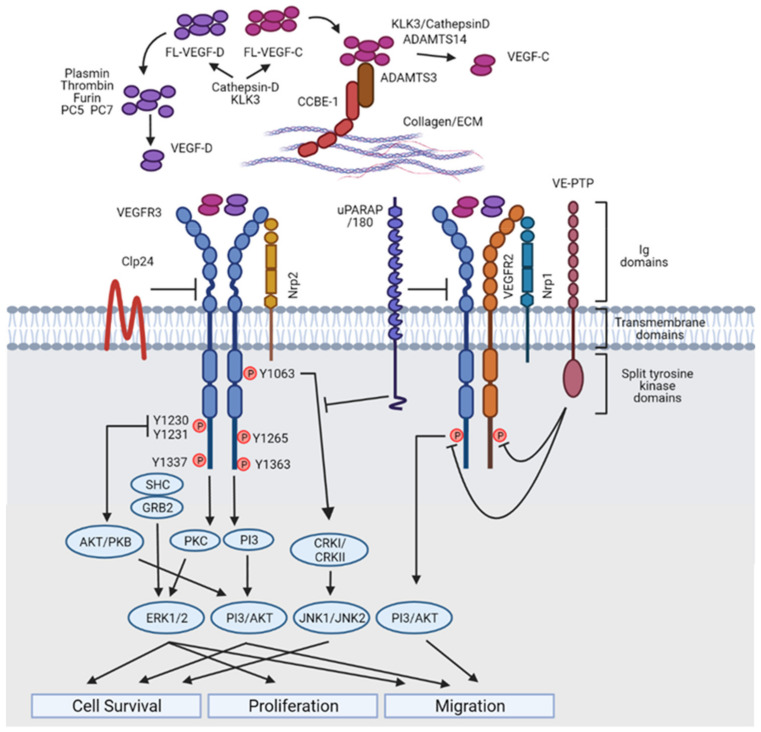
VEGFC/VEGFD/VEGFR signalling in lymphatic endothelial cells. Pro-peptide forms of VEGFC and VEGFD are proteolytically processed to produce active proteins which bind with high affinity to VEGFR2 and VEGFR3. Activation and phosphorylation of VEGFRs results in downstream signalling via JNK1/2, PI3K/AKT and ERK1/2 pathways, regulating cell survival, proliferation and migration. Hetero-dimerisation of VEGFR2 and VEGFR3 results in distinct downstream signalling. A number of co-receptors and accessory signalling molecules regulate the fine tuning of signal magnitude and duration. Created with Biorender.com.

Unlike the well characterised signalling events promoted by VEGFA binding to VEGFR2 in blood vascular endothelial cells, the signalling cascade initiated in lymphatic endothelial cells upon VEGFC binding to VEGFR3 is less well established. Binding of VEGFC or VEGFD to VEGFR3 induces receptor dimerization, autophosphorylation [65,66], recruitment of CRK1/II and GRB2 and activation of downstream signalling pathways including ERK1/2 and protein kinase B (AKT), which act to regulate lymphatic endothelial cell survival, proliferation and migration [67,68] (Figure 2). More recent work comparing signalling initiated by VEGFA and VEGFC in lymphatic endothelial cells demonstrated that VEGFC promoted greater activation of AKT signalling than did VEGFA and, moreover, activated ERK1/2 in a kinetic pattern distinct to that of VEGFA [69]. VEGFC treatment also promoted heterodimerisation between VEGFR2 and VEGFR3, an event not observed in response to VEGFA, and VEGFC initiated AKT activation was dependent on both VEGFR2 and NRP1 [69]. This study also demonstrated that vascular endothelial phosphotyrosine phosphatase (VE-PTP) tempered VEGFC-mediated activation of both VEGFR2 and VEGFR3, together with downstream signalling pathway activity, in lymphatic endothelial cells [69]. While not a great deal is known regarding the transcriptional responses initiated downstream of VEGFC/VEGFR3 signalling, a PROX1/VEGFR3 feedback loop is important both for dialling up VEGFR3 levels in committed PROX1-positive lymphatic endothelial cells and for maintaining PROX1 levels in these cells during developmental lymphangiogenesis [70]. Recent work demonstrated that mitochondrial respiration controls the expression and activity of this feedback loop [71]. Interruption of mitochondrial complex III in mice resulted in the arrest of lymphatic development by mid-gestation. Reduced levels of PROX1 and VEGFR3 were observed in lymphatic endothelial cells of mutant mice and in lymphatic endothelial cells treated with a mitochondrial complex III inhibitor, correlating with altered histone modifications at *Prox1* and *Flt4* loci [71]. These data reveal that mitochondrial sensing of the metabolic microenvironment is important for regulating developmental lymphangiogenesis via control of *Prox1* and *Flt4* expression. Recent work also demonstrated that HOXD10 is activated rapidly following VEGFR3 activation in human lymphatic endothelial cells treated with the selective VEGFR3 ligand VEGFC156S, and is important for lymphatic endothelial migration and tube formation [72]. Deeper insight to the genes activated and repressed downstream of VEGFR3 will further illuminate our understanding of the mechanisms by which VEGFC/VEGFR3 signalling play such a major role in lymphangiogenesis.

## 6. Regulation of VEGFR Signalling in the Lymphatic Vasculature: An Update

**Figure 3:**
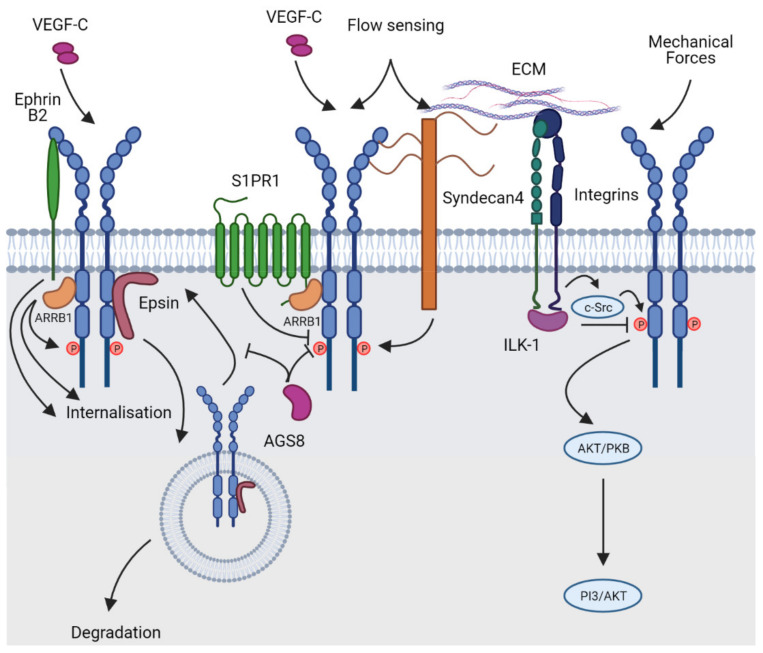
Regulation of VEGFC/VEGFD/VEGFR3 signalling in lymphatic endothelial cells. VEGFC/VEGFD/VEGFR3 signal amplitude is regulated by the endocytosis and subsequent trafficking of internalised receptors. VEGFR3 internalisation from the cell surface is regulated by ephrinB2 and intracellular proteins including ARRB1, epsins 1 and 2 and AGS8. VEGFR3 is also activated by mechanical stimuli including flow and tension in a ligand-independent manner. Interacting proteins that regulate mechanical activation of VEGFR3 include S1PR1, Syndecan 4 and β1-integrin. Created with Biorender.com.

The magnitude of signalling transduced via VEGFR2 and VEGFR3 is subject to regulation at the level of ligand processing and availability, interaction with co-receptors and route of trafficking taken by receptors following ligand-mediated internalisation. While earlier work revealed that VEGFR2 and VEGFR3 internalisation and signalling activity is regulated by molecules including ephrinB2 [73,74], caveolin-1 [75] and epsins [76], recent work has provided further insight into how regulation of VEGFR internalisation impacts signal transduction in various cellular contexts (Figure 3). The β-arrestin ARRB1, a member of the arrestin family of adaptor proteins that regulate G-protein coupled receptor signalling, was recently shown to interact with VEGFR3 in lung microvascular endothelial cells and thereby regulate VEGFC-initiated VEGFR3 internalisation, phosphorylation and downstream signal transduction [77]. The interaction between ARRB1 and VEGFR3 was dependent on VEGFR3 phosphorylation, and depletion of ARRB1 resulted in reduced VEGFR3 phosphorylation, suggesting that interaction with ARRB1 prolongs VEGFR3 signalling [77]. Given that lung microvascular endothelial cells comprise both blood and lymphatic vascular endothelial cells, it would be interesting to further investigate the role of ARRB1 selectively in lymphatic endothelial cells. The link between ARRB1, VEGFR3 and GPCR signalling raised the possibility that VEGFR3 and GPCR signalling pathways may be coupled, potentially via ARRB1, in lymphatic endothelial cells. Evidence for this hypothesis was recently provided in a study demonstrating that the GPCR sphingosine-1-phosphate receptor (S1PR1) acts to dampen laminar shear stress (LSS) dependent VEGFC/VEGFR3 signalling in the lymphatic vasculature, to promote lymphatic vessel quiescence [78]. Intriguingly in this study, S1PR1 deficiency did not block basal VEGFC-initiated VEGFR3 signalling in lymphatic endothelial cells cultured in static conditions but did dampen the elevation in VEGFC/VEGFR3 signalling promoted by LSS [78]. Accordingly, ectopic sprouting of the lymphatic vasculature in mice harbouring a lymphatic specific deletion of *S1pr1* was rescued by the deletion of one allele of *Flt4* encoding VEGFR3 [78]. These data reveal that the mechanical stimulus of flow increases the magnitude of VEGFR3 signalling initiated by VEGFC, though the mechanisms responsible for this effect remain to be fully characterised. Recent studies also demonstrated a role for the ischemia-inducible Gβγ signal regulator, activator of G-protein signalling 8 (AGS8), in regulation of VEGFR3 signal transduction. AGS8 deficient human dermal lymphatic endothelial cells showed decreased proliferation and tube formation when stimulated with VEGFC and decreased phosphorylation of VEGFR3, ERK1/2 and AKT [79]. Analysis of VEGFR3 levels and localisation revealed that reduced levels of VEGFR3 were observed at the cell surface of AGS8 deficient cells, suggesting that trafficking of VEGFR3 to the plasma membrane is regulated by AGS8 [79].

Epsins are a family of ubiquitin-binding adaptor proteins expressed in endothelial cells that regulate clathrin-dependent endocytosis of VEGFRs. Epsin 1 and 2 are important for regulating the endocytosis and degradation of both VEGFR2 [80] and VEGFR3 [76]. Consequently, deficiency of epsins 1 and 2 in endothelial cells results in the inhibition of VEGFR endocytosis and degradation, culminating in elevated VEGFR2 and VEGFR3 signalling. Mice in which epsins 1 and 2 were deleted in the lymphatic vasculature exhibited enlarged initial lymphatics and an arrest in lymphatic vessel valve development [76]. Elevated levels of VEGFR3, phosphorylated VEGFR3 and downstream signalling pathways were observed in lymphatic endothelial cells isolated from epsin deficient mice due to the failure of VEGFR3 internalisation and degradation [76]. The possibility that epsin function might be targeted to elevate VEGFR3 activity in settings of disease where this would be beneficial was recently tested in a mouse model of type II diabetes. In this study, hyperglycemic mice harbouring lymphatic specific deletion of epsin 1 and 2 were subjected to a series of in vivo lymphangiogenesis assays, all of which revealed elevated VEGFC-promoted lymphangiogenesis compared to wild-type diabetic mice [81]. Investigation of the mechanisms underpinning reduced lymphangiogenic activity in wild-type mice demonstrated that high ROS levels initiated by hyperglycemia resulted in elevated epsin levels and increased Src-dependent VEGFR3 phosphorylation, cumulatively increasing VEGFR3 degradation and reducing VEGFR3 signalling activity in lymphatic endothelial cells [81]. These data suggest that inhibiting epsins in the lymphatic vasculature might prove beneficial for the stimulation of lymphangiogenesis in disorders including diabetes, where promoting lymphatic function would aid wound healing and oedema resolution.

While early studies of VEGFR3 signalling revealed that the adaptor protein GRB2 is recruited to phosphorylated VEGFR3 and subsequently activates both AKT and ERK1/2 signalling [68], recent work in zebrafish has shown that loss-of-function mutations in grb2a and grb2b result in severe defects in development of the head and trunk lymphatics, respectively [82]. Zebrafish harbouring a mutation in grb2b exhibited fewer parachordal lymphangioblasts and failed to form a thoracic duct. Accordingly, grb2b deficient mutants exhibited a reduced number of PROX1-positive lymphatic progenitor cells within the PCV. Grb2b was established to regulate VEGFR3 downstream signalling by fine-tuning the balance of MEK/ERK activation [82], a key pathway regulating *Prox1* expression [83]. As a result, mutant fish displayed a decreased number of pERK positive cells in the PCV [82]. This study provided confirmation that grb2b genetically interacts with VEGFR3 to regulate developmental lymphangiogenesis.

While earlier work revealed that membrane proteins including ephrinB2 [73,74], CLP24 [84] and caveolin-1 [75] regulate VEGFR2 and VEGFR3 activity in lymphatic endothelial cells, recent work has identified additional membrane proteins involved in this process. CLEC14A, a type I transmembrane protein belonging to the C-type lectin superfamily, was demonstrated to interact directly with VEGFR3 and thereby modulate VEGFR2 signalling [85]. *Clec14a^−/−^* mice displayed enlarged jugular lymph sacs and dermal lymphatic vessels, together with increased developmental and pathological angiogenesis. While *Clec14a* deficient endothelial cells exhibited reduced levels of VEGFR3, increased levels of VEGFR2 and active downstream signalling components were observed [85]. The mechanisms via which CLEC14A interaction with VEGFR3 result in reduced VEGFR3 and increased VEGFR2 levels remain to be established. The urokinase plasminogen activator receptor-associated protein, uPARAP/Endo180, best recognised for its role as an endocytic collagen receptor [86], was recently shown to play an important role in pathological lymphangiogenesis by regulating heterodimerisation of VEGFR2 and VEGFR3 [87]. Deletion of *uPARAP* in mice resulted in the formation of functional hyperbranched lymphatic vessels in pathological conditions, though did not affect angiogenesis [87]. Mechanistically, uPARAP was shown to interact with both VEGFR2 and VEGFR3 and, as a result, limit the formation of VEGFR2/VEGFR3 heterodimers. Interestingly, uPARAP silencing in lymphatic endothelial cells did not affect VEGFC-initiated phosphorylation of VEGFR2, VEGFR3, AKT or ERK1/2, but did impact the phosphorylation of JNK and paxillin, ultimately resulting in elevated levels of Rac1-GTP [87]. Rac1 inhibition was able to rescue the increased lymphangiogenesis observed in uPARAP deficient mice [87]. In addition to identifying a new binding partner of VEGFR2 and VEGFR3, this study provided valuable insight into signal transduction downstream of VEGFR2/VEGFR3 heterodimers.

An important role for VEGFR2 signalling in the regulation of junctional integrity in the lacteals (specialised lymphatics within the intestinal villi that mediate chylomicron absorption) was recently discovered, further emphasising a key role for VEGFR2 in the lymphatic vasculature. In this study, increased VEGFA bioavailability induced by deletion of the VEGFA receptors VEGFR1, encoded by *Flt1* and *Nrp1* [88], protected mice from diet-induced obesity. Intriguingly, elevated VEGFA bioavailability had the effect of “zippering” lacteal junctions, thereby preventing lipid absorption via the lacteals. This effect of VEGFA on lacteal junctions is in direct contrast to the effect of VEGFA on blood vascular endothelial cell junctions, in which case VEGFA promotes junctional opening and permeability [88]. The reduction of lipid absorption due to lacteal lymphatic endothelial cell junctional tightening opens up the possibility of modulating lacteal junctional integrity therapeutically in order to treat obesity. Further dissection of the roles of VEGFR2 and VEGFR3 signalling in the lymphatic vasculature of distinct tissues will be a fascinating avenue for future investigation.

## 7. Mechanical Regulation of VEGFR Signalling during Lymphatic Vascular Development

The roles of fluid shear stress in regulating vascular development and remodelling are well established [89]. Recent work has provided greater insight into the roles and transmission of shear stress-induced signals important for lymphatic vascular development. VEGFR2 and VEGFR3 are key components of the most extensively characterised mechanosensory complex in endothelial cells, comprising VE-Cadherin, PECAM1, VEGFR2 and VEGFR3 [90,91,92]. This complex resides at cell junctions and transmits flow-initiated signals to changes in cellular architecture that underpin endothelial cell alignment in response to laminar flow. VEGFR3 has been determined to establish the “set-point” at which cells respond to different levels of shear stress [92]. Phosphorylation of VEGFR2 and VEGFR3 is initiated in endothelial cells in response to laminar flow, in a manner independent of VEGFA or VEGFC, but dependent on force transduction via PECAM1 and Src family kinase activation [90,91,92]. Recent work demonstrated that mice harbouring lymphatic vessel selective deletion of *Pecam1* or *Sdc4*, encoding syndecan 4, exhibit defects in lymphatic vessel remodelling and valve morphogenesis due to the inability of lymphatic endothelial cells to align in response to laminar flow [64]. This study found that VEGFR3 phosphorylation was decreased in *Sdn4* deficient cells exposed to flow, and that attributed the defects in lymphatic endothelial cell polarity to elevated levels of the planar cell polarity protein VANGL2 [65]. The possibility exists, given the recent demonstration of a direct interaction between VEGFR3 and SDC4 [63], that defects in PECAM1- and VEGFR3-mediated flow sensing underpin the lymphatic vascular defects in *Sdc4* deficient mice. Similarly, mice deficient in the huge atypical cadherin FAT4 were recently shown to exhibit pronounced lymphatic vascular defects as a result of the failure of FAT4 deficient endothelial cells to polarise appropriately in lymphatic endothelial cells exposed to laminar flow [93]. This study identified an interaction between VEGFR3 and the intracellular domain of FAT4, suggesting that FAT4 might regulate flow-induced lymphatic endothelial cell alignment by modulating activity of the junctional mechanosensory complex [93].

Two recent studies demonstrated that flow-regulated lymphatic endothelial cell alignment is also dependent on VE-Cadherin [94,95]. In the first of these, temporal and tissue specific requirements for VE-Cadherin (encoded by *Cdh5*) in the lymphatic vasculature were demonstrated [94]. While the dermal lymphatic vasculature of postnatal mice did not display striking alterations in pattern or junctional integrity following *Cdh5* deletion, the mesenteric lymphatic vasculature was exquisitely sensitive to *Cdh5* deletion [94]. Features of mesenteric lymphatic vessels in which *Cdh5* was deleted included profound distension, disruption and the abnormal growth of lymphatic endothelial cells over the mesothelial membrane [94]. Altered localisation and increased phosphorylation of VEGFR3 were noted in these mutant vessels, suggesting that VE-cadherin regulates VEGFR3 localisation and signalling capacity, at least in the postnatal mesenteric lymphatic vasculature [94]. Deletion of *Cdh5* in the lymphatic vasculature was also shown to disrupt lymphatic endothelial cell alignment in response to flow and, as a consequence, lymphatic vessel valve development [95], an event regulated by VEGFC signalling [96]. In this case, AKT activation in response to flow was decreased and valve development in VE-cadherin deficient mice could be rescued with a small molecule activator of AKT [95]. Whether AKT activation in this context was dependent on VEGFR3 was not established, though previous work demonstrated that AKT is prominently activated downstream of VEGFR3 [67,68]. Further elaboration of the signalling events resulting in VEGFR3 activation and downstream signal transduction initiated by flow in lymphatic endothelial cells await future studies.

In addition to flow, mechanical signals including tension forces have also been shown to induce VEGFC-independent VEGFR3 signalling [97]. An elegant early study demonstrating the effects of modulating interstitial fluid levels on lymphangiogenesis revealed that increased interstitial fluid levels and resultant lymphatic endothelial cell stretching promoted VEGFR3 phosphorylation in a manner dependent on β1-integrin activation [97]. A direct interaction between VEGFR3 and β1-integrin was documented in this study and lymphatic vascular defects were observed in β1-integrin deficient mice, demonstrating the importance of the β1-integrin for VEGFR3 activation during developmental lymphangiogenesis [97]. More recent work showed that the integrin-linked kinase (ILK) acts in a cell-autonomous manner to regulate VEGFR3 signalling and that loss of ILK resulted in increased signalling downstream of VEGFR3, increased LEC proliferation and lymphatic vascular overgrowth [98]. This study demonstrated that ILK interrupts the interaction of VEGFR3 with β1-integrin, acting to restrain VEGFR3 activity [98]. Whether the involvement of VEGFR3 in sensing tension-induced mechanical signals and transducing these to changes in cell proliferation depends on VEGFR3 being a component of the junctional mechanosensory complex, or a distinct complex involving β1-integrin, and potentially additional mechanosensory components, will be an interesting avenue of future investigation.

## 8. VEGFC/VEGFD/VEGFR3 Signalling in Lymphatic Vascular Disease

Recently published work has highlighted the increasing number of human pathologies in which the lymphatic vasculature is implicated and which stand to benefit from therapeutic modulation of VEGFA/VEGFC/VEGFD/VEGFR2/VEGFR3 signalling [4,5]. The apparently selective response of the meningeal and lacteal lymphatics to VEGFC/D/R3 modulating agents during adulthood may provide the opportunity to intervene in this pathway therapeutically with minimal off-target effects. Recent studies in mice have highlighted the benefits of promoting lymphangiogenesis of the meningeal lymphatic vessels in increasing the efficacy of checkpoint inhibitor treatment for glioblastoma [99], and promoting the clearance of amyloid β in a mouse model of Alzheimer’s disease via immunotherapy with anti-amyloid β (Aβ) antibodies [100]. In a similar vein, delivery of VEGFC to cardiac tissue has been demonstrated to promote lymphangiogenesis and the clearance of tissue fluid and inflammation, reducing fibrosis and improving regeneration in mouse [101,102], rat [103] and zebrafish [104] models of cardiac injury. Intriguingly, in zebrafish, the dependence on VEGFC/VEGFD for cardiac regeneration was found to be distinctly dependent on the type of injury inflicted [104]. In contrast to these settings where promoting VEGFC/VEGFR3-mediated lymphangiogenesis is beneficial, diseases including lymphangioleiomyomatosis (LAM) [105], Gorham-Stout Disease (in which the lymphatics invade bone) [106] and lymphatic malformations driven by PIK3CA mutations [107] are characterised by elevated VEGFC- and VEGFD-initiated VEGFR3 signalling. In these cases, inhibition of signalling via this axis, either by VEGFC/D traps or VEGFR3 small molecule inhibitors, stands to be of therapeutic benefit. A soluble form of VEGFR3 that blocks the interaction of VEGFC and VEGFD with VEGFR2 and VEGFR3 is currently in clinical trial for wet age-related macular degeneration and diabetic macular oedema, both of which are characterised by the development of tortuous and leaky blood vessels in the eye [108]. Finely tuned modulation of VEGFR2 and VEGFR3 activity might also be achieved by targeting specific VEGFR2- or VEGFR3-interacting proteins such as epsins, uPARAP or ILK. Defining the fundamental molecular basis of VEGF family-initiated VEGFR2 and VEGFR3 signal transduction will be crucial to underpin the development of novel therapeutics able to treat pathologies involving the lymphatic vasculature.

## 9. Conclusions and Future Directions

Our knowledge of the mechanisms regulating both the spatiotemporal restriction and amplitude of signalling initiated by VEGF family members and their receptors has advanced substantially over the last five years. The identification of new proteases that proteolytically activate VEGFC and VEGFD, together with their cellular sources and matrix components responsible for regulating ligand cleavage and/or retention, have vastly improved our understanding of the mechanisms regulating patterning of the lymphatic vasculature during development. It will be intriguing in future work to investigate how similar, or distinct, the factors regulating lymphatic patterning are across different tissues and in settings of pathology. We have also gained new insight into the co-receptors and interacting proteins that modulate VEGF ligand binding and receptor activation, together with the intracellular adaptor proteins that control receptor internalisation and downstream trafficking events. There remains much to learn on this front, particularly in terms of the downstream trafficking events that regulate the duration of VEGFR3 signalling as well as receptor availability and localisation. There is also more to learn regarding the mechanisms by which signalling induced by VEGFR2/VEGFR3 heterodimers is distinct from that induced by VEGFR2 or VEGFR3 homodimers and how downstream signalling pathways are activated in lymphatic, compared to blood vascular, endothelial cells. The transmission of mechanical signals via VEGF receptors is another expanding field of research warranting further investigation. Detailed knowledge of each aspect of signal transduction mediated by VEGF family ligands and their receptors will provide new opportunities to modulate these signalling events and thereby provide improved therapeutics for the wide range of pathologies in which the lymphatic vasculature is involved.

## Data Availability

Not applicable.

## References

[1] Tammela T., Alitalo K. (2010). Lymphangiogenesis: Molecular mechanisms and future promise. Cell.

[2] Petrova T.V., Koh G.Y. (2018). Organ-specific lymphatic vasculature: From development to pathophysiology. J. Exp. Med..

[3] Lord R.S. (1968). The white veins: Conceptual difficulties in the history of the lymphatics. Med. Hist..

[4] Petrova T.V., Koh G.Y. (2020). Biological functions of lymphatic vessels. Science.

[5] Oliver G., Kipnis J., Randolph G.J., Harvey N.L. (2020). The Lymphatic Vasculature in the 21(st) Century: Novel Functional Roles in Homeostasis and Disease. Cell.

[6] Trevaskis N.L., Kaminskas L.M., Porter C.J. (2015). From sewer to saviour—Targeting the lymphatic system to promote drug exposure and activity. Nat. Rev. Drug. Discov..

[7] Feeney O.M., Gracia G., Brundel D.H.S., Trevaskis N.L., Cao E., Kaminskas L.M., Porter C.J.H. (2020). Lymph-directed immunotherapy—Harnessing endogenous lymphatic distribution pathways for enhanced therapeutic outcomes in cancer. Adv. Drug Deliv. Rev..

[8] Baluk P., Fuxe J., Hashizume H., Romano T., Lashnits E., Butz S., Vestweber D., Corada M., Molendini C., Dejana E. (2007). Functionally specialized junctions between endothelial cells of lymphatic vessels. Exp. Med..

[9] Van der Putte S.C. (1975). The early development of the lymphatic system in mouse embryos. Acta Morphol. Neerl. Scand..

[10] Bazigou E., Makinen T. (2013). Flow control in our vessels: Vascular valves make sure there is no way back. Cell. Mol. Life Sci..

[11] Muthuchamy M., Zawieja D. (2008). Molecular regulation of lymphatic contractility. Ann. N. Y. Acad. Sci..

[12] Bachmann S.B., Gsponer D., Montoya-Zegarra J.A., Schneider M., Scholkmann F., Tacconi C., Noerrelykke S.F., Proulx S.T., Detmar M. (2019). A Distinct Role of the Autonomic Nervous System in Modulating the Function of Lymphatic Vessels under Physiological and Tumor-Draining Conditions. Cell Rep..

[13] Choe K., Jang J.Y., Park I., Kim Y., Ahn S., Park D.Y., Hong Y.K., Alitalo K., Koh G.Y., Kim P. (2015). Intravital imaging of intestinal lacteals unveils lipid drainage through contractility. J. Clin. Investig..

[14] Brouillard P., Boon L., Vikkula M. (2014). Genetics of lymphatic anomalies. J. Clin. Investig..

[15] Wigle J.T., Oliver G. (1999). Prox1 function is required for the development of the murine lymphatic system. Cell.

[16] Francois M., Short K., Secker G.A., Combes A., Schwarz Q., Davidson T.L., Smyth I., Hong Y.K., Harvey N.L., Koopman P. (2012). Segmental territories along the cardinal veins generate lymph sacs via a ballooning mechanism during embryonic lymphangiogenesis in mice. Dev. Biol..

[17] Yang Y., Garcia-Verdugo J.M., Soriano-Navarro M., Srinivasan R.S., Scallan J.P., Singh M.K., Epstein J.A., Oliver G. (2012). Lymphatic endothelial progenitors bud from the cardinal vein and intersomitic vessels in mammalian embryos. Blood.

[18] Hagerling R., Pollmann C., Andreas M., Schmidt C., Nurmi H., Adams R.H., Alitalo K., Andresen V., Schulte-Merker S., Kiefer F. (2013). A novel multistep mechanism for initial lymphangiogenesis in mouse embryos based on ultramicroscopy. EMBO J..

[19] Pichol-Thievend C., Betterman K.L., Liu X., Ma W., Skoczylas R., Lesieur E., Bos F.L., Schulte D., Schulte-Merker S., Hogan B.M. (2018). A blood capillary plexus-derived population of progenitor cells contributes to genesis of the dermal lymphatic vasculature during embryonic development. Development.

[20] Karkkainen M.J., Haiko P., Sainio K., Partanen J., Taipale J., Petrova T.V., Jeltsch M., Jackson D.G., Talikka M., Rauvala H. (2004). Vascular endothelial growth factor C is required for sprouting of the first lymphatic vessels from embryonic veins. Nat. Immunol..

[21] Hogan B.M., Bos F.L., Bussmann J., Witte M., Chi N.C., Duckers H.J., Schulte-Merker S. (2009). Ccbe1 is required for embryonic lymphangiogenesis and venous sprouting. Nat. Genet..

[22] Bos F.L., Caunt M., Peterson-Maduro J., Planas-Paz L., Kowalski J., Karpanen T., van Impel A., Tong R., Ernst J.A., Korving J. (2011). CCBE1 is essential for mammalian lymphatic vascular development and enhances the lymphangiogenic effect of vascular endothelial growth factor-C in vivo. Circ. Res..

[23] Bui H.M., Enis D., Robciuc M.R., Nurmi H.J., Cohen J., Chen M., Yang Y., Dhillon V., Johnson K., Zhang H. (2016). Proteolytic activation defines distinct lymphangiogenic mechanisms for VEGFC and VEGFD. Clin. Invest..

[24] Janssen L., Dupont L., Bekhouche M., Noel A., Leduc C., Voz M., Peers B., Cataldo D., Apte S.S., Dubail J. (2016). ADAMTS3 activity is mandatory for embryonic lymphangiogenesis and regulates placental angiogenesis. Angiogenesis.

[25] Jeltsch M., Jha S.K., Tvorogov D., Anisimov A., Leppanen V.M., Holopainen T., Kivela R., Ortega S., Karpanen T., Alitalo K. (2014). CCBE1 enhances lymphangiogenesis via a disintegrin and metalloprotease with thrombospondin motifs-3-mediated vascular endothelial growth factor-C activation. Circulation.

[26] Le Guen L., Karpanen T., Schulte D., Harris N.C., Koltowska K., Roukens G., Bower N.I., van Impel A., Stacker S.A., Achen M.G. (2014). Ccbe1 regulates Vegfc-mediated induction of Vegfr3 signaling during embryonic lymphangiogenesis. Development.

[27] Koltowska K., Lagendijk A.K., Pichol-Thievend C., Fischer J.C., Francois M., Ober E.A., Yap A.S., Hogan B.M. (2015). Vegfc Regulates Bipotential Precursor Division and Prox1 Expression to Promote Lymphatic Identity in Zebrafish. Cell Rep..

[28] Baldwin M.E., Halford M.M., Roufail S., Williams R.A., Hibbs M.L., Grail D., Kubo H., Stacker S.A., Achen M.G. (2005). Vascular endothelial growth factor D is dispensable for development of the lymphatic system. Mol. Cell. Biol..

[29] Paquet-Fifield S., Levy S.M., Sato T., Shayan R., Karnezis T., Davydova N., Nowell C.J., Roufail S., Ma G.Z., Zhang Y.F. (2013). Vascular endothelial growth factor-d modulates caliber and function of initial lymphatics in the dermis. J. Investig. Dermatol..

[30] Bower N.I., Vogrin A.J., le Guen L., Chen H., Stacker S.A., Achen M.G., Hogan B.M. (2017). Vegfd modulates both angiogenesis and lymphangiogenesis during zebrafish embryonic development. Development.

[31] Vogrin A.J., Bower N.I., Gunzburg M.J., Roufail S., Okuda K.S., Paterson S., Headey S.J., Stacker S.A., Hogan B.M., Achen M.G. (2019). Evolutionary Differences in the Vegf/Vegfr Code Reveal Organotypic Roles for the Endothelial Cell Receptor Kdr in Developmental Lymphangiogenesis. Cell Rep..

[32] Dumont D.J., Jussila L., Taipale J., Lymboussaki A., Mustonen T., Pajusola K., Breitman M., Alitalo K. (1998). Cardiovascular failure in mouse embryos deficient in VEGF receptor-3. Science.

[33] Karkkainen M.J., Saaristo A., Jussila L., Karila K.A., Lawrence E.C., Pajusola K., Bueler H., Eichmann A., Kauppinen R., Kettunen M.I. (2001). A model for gene therapy of human hereditary lymphedema. Proc. Natl. Acad. Sci. USA.

[34] Zhang L., Zhou F., Han W., Shen B., Luo J., Shibuya M., He Y. (2010). VEGFR-3 ligand-binding and kinase activity are required for lymphangiogenesis but not for angiogenesis. Cell Res..

[35] Zhang Y., Ulvmar M.H., Stanczuk L., Martinez-Corral I., Frye M., Alitalo K., Makinen T. (2018). Heterogeneity in VEGFR3 levels drives lymphatic vessel hyperplasia through cell-autonomous and non-cell-autonomous mechanisms. Nat. Commun..

[36] Veikkola T., Jussila L., Makinen T., Karpanen T., Jeltsch M., Petrova T.V., Kubo H., Thurston G., McDonald D.M., Achen M.G. (2001). Signalling via vascular endothelial growth factor receptor-3 is sufficient for lymphangiogenesis in transgenic mice. EMBO J..

[37] Karaman S., Leppanen V.M., Alitalo K. (2018). Vascular endothelial growth factor signaling in development and disease. Development.

[38] Makinen T., Jussila L., Veikkola T., Karpanen T., Kettunen M.I., Pulkkanen K.J., Kauppinen R., Jackson D.G., Kubo H., Nishikawa S. (2001). Inhibition of lymphangiogenesis with resulting lymphedema in transgenic mice expressing soluble VEGF receptor-3. Nat. Med..

[39] Karpanen T., Wirzenius M., Makinen T., Veikkola T., Haisma H.J., Achen M.G., Stacker S.A., Pytowski B., Yla-Herttuala S., Alitalo K. (2006). Lymphangiogenic growth factor responsiveness is modulated by postnatal lymphatic vessel maturation. Am. J. Pathol..

[40] Nurmi H., Saharinen P., Zarkada G., Zheng W., Robciuc M.R., Alitalo K. (2015). VEGF-C is required for intestinal lymphatic vessel maintenance and lipid absorption. EMBO Mol. Med..

[41] Antila S., Karaman S., Nurmi H., Airavaara M., Voutilainen M.H., Mathivet T., Chilov D., Li Z., Koppinen T., Park J.H. (2017). Development and plasticity of meningeal lymphatic vessels. Exp. Med..

[42] Achen M.G., Stacker S.A. (1998). The vascular endothelial growth factor family; Proteins which guide the development of the vasculature. Int. J. Exp. Pathol..

[43] Simons M., Gordon E., Claesson-Welsh L. (2016). Mechanisms and regulation of endothelial VEGF receptor signalling. Nat. Rev..

[44] Nagy J.A., Vasile E., Feng D., Sundberg C., Brown L.F., Detmar M.J., Lawitts J.A., Benjamin L., Tan X., Manseau E.J. (2002). Vascular permeability factor/vascular endothelial growth factor induces lymphangiogenesis as well as angiogenesis. Exp. Med..

[45] Wirzenius M., Tammela T., Uutela M., He Y., Odorisio T., Zambruno G., Nagy J.A., Dvorak H.F., Yla-Herttuala S., Shibuya M. (2007). Distinct vascular endothelial growth factor signals for lymphatic vessel enlargement and sprouting. Exp. Med..

[46] Rissanen T.T., Markkanen J.E., Gruchala M., Heikura T., Puranen A., Kettunen M.I., Kholova I., Kauppinen R.A., Achen M.G., Stacker S.A. (2003). VEGF-D is the strongest angiogenic and lymphangiogenic effector among VEGFs delivered into skeletal muscle via adenoviruses. Circ. Res..

[47] Tammela T., Zarkada G., Wallgard E., Murtomaki A., Suchting S., Wirzenius M., Waltari M., Hellstrom M., Schomber T., Peltonen R. (2008). Blocking VEGFR-3 suppresses angiogenic sprouting and vascular network formation. Nature.

[48] Dixelius J., Makinen T., Wirzenius M., Karkkainen M.J., Wernstedt C., Alitalo K., Claesson-Welsh L. (2003). Ligand-induced vascular endothelial growth factor receptor-3 (VEGFR-3) heterodimerization with VEGFR-2 in primary lymphatic endothelial cells regulates tyrosine phosphorylation sites. J. Biol. Chem..

[49] Nilsson I., Bahram F., Li X., Gualandi L., Koch S., Jarvius M., Soderberg O., Anisimov A., Kholova I., Pytowski B. (2010). VEGF receptor 2/-3 heterodimers detected in situ by proximity ligation on angiogenic sprouts. EMBO J..

[50] Kunnapuu J., Bokharaie H., Jeltsch M. (2021). Proteolytic Cleavages in the VEGF Family: Generating Diversity among Angiogenic VEGFs, Essential for the Activation of Lymphangiogenic VEGFs. Biology.

[51] Wang G., Muhl L., Padberg Y., Dupont L., Peterson-Maduro J., Stehling M., le Noble F., Colige A., Betsholtz C., Schulte-Merker S. (2020). Specific fibroblast subpopulations and neuronal structures provide local sources of Vegfc-processing components during zebrafish lymphangiogenesis. Nat. Commun..

[52] McColl B.K., Baldwin M.E., Roufail S., Freeman C., Moritz R.L., Simpson R.J., Alitalo K., Stacker S.A., Achen M.G. (2003). Plasmin activates the lymphangiogenic growth factors VEGF-C and VEGF-D. Exp. Med..

[53] Lim L., Bui H., Farrelly O., Yang J., Li L., Enis D., Ma W., Chen M., Oliver G., Welsh J.D. (2019). Hemostasis stimulates lymphangiogenesis through release and activation of VEGFC. Blood.

[54] Jha S.K., Rauniyar K., Chronowska E., Mattonet K., Maina E.W., Koistinen H., Stenman U.H., Alitalo K., Jeltsch M. (2019). KLK3/PSA and cathepsin D activate VEGF-C and VEGF-D. elife.

[55] McColl B.K., Paavonen K., Karnezis T., Harris N.C., Davydova N., Rothacker J., Nice E.C., Harder K.W., Roufail S., Hibbs M.L. (2007). Proprotein convertases promote processing of VEGF-D, a critical step for binding the angiogenic receptor VEGFR-2. FASEB J..

[56] Chaudhury S., Okuda K.S., Koltowska K., Lagendijk A.K., Paterson S., Baillie G.J., Simons C., Smith K.A., Hogan B.M., Bower N.I. (2020). Localised Collagen2a1 secretion supports lymphatic endothelial cell migration in the zebrafish embryo. Development.

[57] Leppanen V.M., Tvorogov D., Kisko K., Prota A.E., Jeltsch M., Anisimov A., Markovic-Mueller S., Stuttfeld E., Goldie K.N., Ballmer-Hofer K. (2013). Structural and mechanistic insights into VEGF receptor 3 ligand binding and activation. Proc. Natl. Acad. Sci. USA.

[58] Herzog Y., Kalcheim C., Kahane N., Reshef R., Neufeld G. (2001). Differential expression of neuropilin-1 and neuropilin-2 in arteries and veins. Mech. Dev..

[59] Bouvree K., Brunet I., del Toro R., Gordon E., Prahst C., Cristofaro B., Mathivet T., Xu Y., Soueid J., Fortuna V. (2012). Semaphorin3A, Neuropilin-1, and PlexinA1 are required for lymphatic valve formation. Circ. Res..

[60] Jurisic G., Maby-El Hajjami H., Karaman S., Ochsenbein A.M., Alitalo A., Siddiqui S.S., Ochoa Pereira C., Petrova T.V., Detmar M. (2012). An unexpected role of semaphorin3a-neuropilin-1 signaling in lymphatic vessel maturation and valve formation. Circ. Res..

[61] Yuan L., Moyon D., Pardanaud L., Breant C., Karkkainen M.J., Alitalo K., Eichmann A. (2002). Abnormal lymphatic vessel development in neuropilin 2 mutant mice. Development.

[62] Yan H., Zhang C., Wang Z., Tu T., Duan H., Luo Y., Feng J., Liu F., Yan X. (2017). CD146 is required for VEGF-C-induced lymphatic sprouting during lymphangiogenesis. Sci. Rep..

[63] Johns S.C., Yin X., Jeltsch M., Bishop J.R., Schuksz M., El Ghazal R., Wilcox-Adelman S.A., Alitalo K., Fuster M.M. (2016). Functional Importance of a Proteoglycan Coreceptor in Pathologic Lymphangiogenesis. Circ. Res..

[64] Wang Y., Baeyens N., Corti F., Tanaka K., Fang J.S., Zhang J., Jin Y., Coon B., Hirschi K.K., Schwartz M.A. (2016). Syndecan 4 controls lymphatic vasculature remodeling during mouse embryonic development. Development.

[65] Joukov V., Pajusola K., Kaipainen A., Chilov D., Lahtinen I., Kukk E., Saksela O., Kalkkinen N., Alitalo K. (1996). A novel vascular endothelial growth factor, VEGF-C, is a ligand for the Flt4 (VEGFR-3) and KDR (VEGFR-2) receptor tyrosine kinases. EMBO J..

[66] Achen M.G., Jeltsch M., Kukk E., Makinen T., Vitali A., Wilks A.F., Alitalo K., Stacker S.A. (1998). Vascular endothelial growth factor D (VEGF-D) is a ligand for the tyrosine kinases VEGF receptor 2 (Flk1) and VEGF receptor 3 (Flt4). Proc. Natl. Acad. Sci. USA.

[67] Makinen T., Veikkola T., Mustjoki S., Karpanen T., Catimel B., Nice E.C., Wise L., Mercer A., Kowalski H., Kerjaschki D. (2001). Isolated lymphatic endothelial cells transduce growth, survival and migratory signals via the VEGF-C/D receptor VEGFR-3. EMBO J..

[68] Salameh A., Galvagni F., Bardelli M., Bussolino F., Oliviero S. (2005). Direct recruitment of CRK and GRB2 to VEGFR-3 induces proliferation, migration, and survival of endothelial cells through the activation of ERK, AKT, and JNK pathways. Blood.

[69] Deng Y., Zhang X., Simons M. (2015). Molecular controls of lymphatic VEGFR3 signaling. Arterioscler. Thromb. Vasc. Biol..

[70] Srinivasan R.S., Escobedo N., Yang Y., Interiano A., Dillard M.E., Finkelstein D., Mukatira S., Gil H.J., Nurmi H., Alitalo K. (2014). The Prox1-Vegfr3 feedback loop maintains the identity and the number of lymphatic endothelial cell progenitors. Genes Dev..

[71] Ma W., Gil H.J., Liu X., Diebold L.P., Morgan M.A., Oxendine-Burns M.J., Gao P., Chandel N.S., Oliver G. (2021). Mitochondrial respiration controls the Prox1-Vegfr3 feedback loop during lymphatic endothelial cell fate specification and maintenance. Sci. Adv..

[72] Klein S., Dieterich L.C., Mathelier A., Chong C., Sliwa-Primorac A., Hong Y.K., Shin J.W., Lizio M., Itoh M., Kawaji H. (2016). DeepCAGE transcriptomics identify HOXD10 as a transcription factor regulating lymphatic endothelial responses to VEGF-C. J. Cell Sci..

[73] Sawamiphak S., Seidel S., Essmann C.L., Wilkinson G.A., Pitulescu M.E., Acker T., Acker-Palmer A. (2010). Ephrin-B2 regulates VEGFR2 function in developmental and tumour angiogenesis. Nature.

[74] Wang Y., Nakayama M., Pitulescu M.E., Schmidt T.S., Bochenek M.L., Sakakibara A., Adams S., Davy A., Deutsch U., Luthi U. (2010). Ephrin-B2 controls VEGF-induced angiogenesis and lymphangiogenesis. Nature.

[75] Galvagni F., Anselmi F., Salameh A., Orlandini M., Rocchigiani M., Oliviero S. (2007). Vascular endothelial growth factor receptor-3 activity is modulated by its association with caveolin-1 on endothelial membrane. Biochemistry.

[76] Liu X., Pasula S., Song H., Tessneer K.L., Dong Y., Hahn S., Yago T., Brophy M.L., Chang B., Cai X. (2014). Temporal and spatial regulation of epsin abundance and VEGFR3 signaling are required for lymphatic valve formation and function. Sci. Signal..

[77] Ma Z., Yu Y.R., Badea C.T., Kovacs J.J., Xiong X., Comhair S., Piantadosi C.A., Rajagopal S. (2019). Vascular Endothelial Growth Factor Receptor 3 Regulates Endothelial Function Through beta-Arrestin 1. Circulation.

[78] Geng X., Yanagida K., Akwii R.G., Choi D., Chen L., Ho Y., Cha B., Mahamud M.R., Berman de Ruiz K., Ichise H. (2020). S1PR1 regulates the quiescence of lymphatic vessels by inhibiting laminar shear stress-dependent VEGF-C signaling. JCI Insight.

[79] Sakima M., Hayashi H., Mamun A.A., Sato M. (2018). VEGFR-3 signaling is regulated by a G-protein activator, activator of G-protein signaling 8, in lymphatic endothelial cells. Exp. Cell Res..

[80] Pasula S., Cai X., Dong Y., Messa M., McManus J., Chang B., Liu X., Zhu H., Mansat R.S., Yoon S.J. (2012). Endothelial epsin deficiency decreases tumor growth by enhancing VEGF signaling. J. Clin. Investig..

[81] Wu H., Rahman H.N.A., Dong Y., Liu X., Lee Y., Wen A., To K.H., Xiao L., Birsner A.E., Bazinet L. (2018). Epsin deficiency promotes lymphangiogenesis through regulation of VEGFR3 degradation in diabetes. J. Clin. Investig..

[82] Mauri C., van Impel A., Mackay E.W., Schulte-Merker S. (2021). The adaptor protein Grb2b is an essential modulator for lympho-venous sprout formation in the zebrafish trunk. Angiogenesis.

[83] Deng Y., Atri D., Eichmann A., Simons M. (2013). Endothelial ERK signaling controls lymphatic fate specification. J. Clin. Investig..

[84] Saharinen P., Helotera H., Miettinen J., Norrmen C., D’Amico G., Jeltsch M., Langenberg T., Vandevelde W., Ny A., Dewerchin M. (2010). Claudin-like protein 24 interacts with the VEGFR-2 and VEGFR-3 pathways and regulates lymphatic vessel development. Genes Dev..

[85] Lee S., Rho S.S., Park H., Park J.A., Kim J., Lee I.K., Koh G.Y., Mochizuki N., Kim Y.M., Kwon Y.G. (2017). Carbohydrate-binding protein CLEC14A regulates VEGFR-2- and VEGFR-3-dependent signals during angiogenesis and lymphangiogenesis. J. Clin. Investig..

[86] Engelholm L.H., Ingvarsen S., Jurgensen H.J., Hillig T., Madsen D.H., Nielsen B.S., Behrendt N. (2009). The collagen receptor uPARAP/Endo180. Front. Biosci..

[87] Durre T., Morfoisse F., Erpicum C., Ebroin M., Blacher S., Garcia-Caballero M., Deroanne C., Louis T., Balsat C., van de Velde M. (2018). uPARAP/Endo180 receptor is a gatekeeper of VEGFR-2/VEGFR-3 heterodimerisation during pathological lymphangiogenesis. Nat. Commun..

[88] Zhang F., Zarkada G., Han J., Li J., Dubrac A., Ola R., Genet G., Boyé K., Michon P., Künzel S.E. (2018). Lacteal junction zippering protects against diet-induced obesity. Science.

[89] Gordon E., Schimmel L., Frye M. (2020). The Importance of Mechanical Forces for in vitro Endothelial Cell Biology. Front. Physiol..

[90] Tzima E., Irani-Tehrani M., Kiosses W.B., Dejana E., Schultz D.A., Engelhardt B., Cao G., Delisser H.M., Schwartz M.A. (2005). A mechanosensory complex that mediates the endothelial cell response to fluid shear stress. Nat. Cell Biol..

[91] Coon B.G., Baeyens N., Han J., Budatha M., Ross T.D., Fang J.S., Yun S., Thomas J.L., Schwartz M.A. (2015). Intramembrane binding of VE-cadherin to VEGFR2 and VEGFR3 assembles the endothelial mechanosensory complex. J. Cell Biol..

[92] Baeyens N., Nicoli S., Coon B.G., Ross T., Dries K.V.D., Han J., Lauridsen H.M., Mejean C.O., Eichmann A., Thomas J.-L. (2015). Vascular remodeling is governed by a VEGFR3-dependent fluid shear stress set point. eLife.

[93] Betterman K.L., Sutton D.L., Secker G.A., Kazenwadel J., Oszmiana A., Lim L., Miura N., Sorokin L., Hogan B.M., Kahn M.L. (2020). Atypical cadherin FAT4 orchestrates lymphatic endothelial cell polarity in response to flow. J. Clin. Investig..

[94] Hägerling R., Hoppe E., Dierkes C., Stehling M., Makinen T., Butz S., Vestweber D., Kiefer F. (2018). Distinct roles of VE-cadherin for development and maintenance of specific lymph vessel beds. EMBO J..

[95] Yang Y., Cha B., Motawe Z.Y., Srinivasan R.S., Scallan J.P. (2019). VE-Cadherin Is Required for Lymphatic Valve Formation and Maintenance. Cell Rep..

[96] Cha B., Ho Y.-C., Geng X., Mahamud R., Chen L., Kim Y., Choi D., Kim T.H., Randolph G.J., Cao X. (2020). YAP and TAZ maintain PROX1 expression in the developing lymphatic and lymphovenous valves in response to VEGF-C signaling. Development.

[97] Planas-Paz L., Strilić B., Goedecke A., Breier G., Fässler R., Lammert E. (2011). Mechanoinduction of lymph vessel expansion. EMBO J..

[98] Urner S., Planas-Paz L., Hilger L.S., Henning C., Branopolski A., Kelly-Goss M., Stanczuk L., Pitter B., Montanez E., Peirce S. (2018). Identification of ILK as a critical regulator of VEGFR 3 signalling and lymphatic vascular growth. EMBO J..

[99] Song E., Mao T., Dong H., Boisserand L.S.B., Antila S., Bosenberg M., Alitalo K., Thomas J.L., Iwasaki A. (2020). VEGF-C-driven lymphatic drainage enables immunosurveillance of brain tumours. Nature.

[100] Da Mesquita S., Papadopoulos Z., Dykstra T., Brase L., Farias F.G., Wall M., Jiang H., Kodira C.D., de Lima K.A., Herz J. (2021). Meningeal lymphatics affect microglia responses and anti-Abeta immunotherapy. Nature.

[101] Klotz L., Norman S., Vieira J., Masters M., Rohling M., Dubé K.N., Bollini S., Matsuzaki F., Carr C.A., Riley P.R. (2015). Cardiac lymphatics are heterogeneous in origin and respond to injury. Nat. Cell Biol..

[102] Vieira J., Norman S., del Campo C.V., Cahill T.J., Barnette D.N., Gunadasa-Rohling M., Johnson L., Greaves D.R., Carr C.A., Jackson D.G. (2018). The cardiac lymphatic system stimulates resolution of inflammation following myocardial infarction. J. Clin. Investig..

[103] Henri O., Pouehe C., Houssari M., Galas L., Nicol L., Edwards-Lévy F., Henry J.-P., Dumesnil A., Boukhalfa I., Banquet S. (2016). Selective Stimulation of Cardiac Lymphangiogenesis Reduces Myocardial Edema and Fibrosis Leading to Improved Cardiac Function Following Myocardial Infarction. Circulation.

[104] Vivien C.J., Pichol-Thievend C., Sim C.B., Smith J.B., Bower N.I., Hogan B., Hudson J.E., Francois M., Porrello E.R. (2019). Vegfc/d-dependent regulation of the lymphatic vasculature during cardiac regeneration is influenced by injury context. npj Regen. Med..

[105] Nishino K., Yoshimatsu Y., Muramatsu T., Sekimoto Y., Mitani K., Kobayashi E., Okamoto S., Ebana H., Okada Y., Kurihara M. (2021). Isolation and characterisation of lymphatic endothelial cells from lung tissues affected by lymphangioleiomyomatosis. Sci. Rep..

[106] Hominick D., Silva A., Khurana N., Liu Y., Dechow P.C., Feng J.Q., Pytowski B., Rutkowski J., Alitalo K., Dellinger M.T. (2018). VEGF-C promotes the development of lymphatics in bone and bone loss. eLife.

[107] Martinez-Corral I., Zhang Y., Petkova M., Ortsäter H., Sjöberg S., Castillo S.D., Brouillard P., Libbrecht L., Saur D., Graupera M. (2020). Blockade of VEGF-C signaling inhibits lymphatic malformations driven by oncogenic PIK3CA mutation. Nat. Commun..

[108] Stacker S.A., Achen M.G. (2018). Emerging Roles for VEGF-D in Human Disease. Biomolecules.

